# Development and validation of the Nursing Process Evaluation Tool (NPET): a multidimensional instrument for assessing the quality of AI-generated nursing documentation

**DOI:** 10.1186/s12912-025-04068-8

**Published:** 2025-11-21

**Authors:** Mohammad Othman Abudari, Manar Abu-abbas, Mohammad Al-Ma’ani, Mutaz foad Alradaydeh, Hamza Alduraidi

**Affiliations:** 1 School of Nursing, The University of Jordan, Aqaba, Jordan; 2 School of Nursing, Yarmouk University, Irbid, Jordan; 3 College of Nursing, Al-Balqa Applied University, Al-Salt, Jordan; 4 School of Nursing, The University of Jordan, Amman, Jordan

**Keywords:** Nursing documentation, Artificial intelligence, Psychometrics, Validation, Generative AI models, Instrument development.

## Abstract

**Background:**

The integration of generative artificial intelligence (AI) tools into nursing practice has accelerated documentation processes but it has also raised concerns regarding the completeness, accuracy, and clinical safety of AI-generated care plans. Despite the growing use of tools like ChatGPT, Gemini, and PopAI in clinical and academic settings, no validated instrument currently exists to assess the quality of such documentation across the nursing process.

**Objective:**

This study aimed to develop and validate the Nursing Process Evaluation Tool (NPET), a multidimensional instrument designed to assess the quality of AI-generated nursing documentation within the ADPIE (Assessment, Diagnosis, Planning, Implementation, Evaluation) framework.

**Methods:**

A two-phase cross-sectional study was conducted. Phase I focused on item development and content validation via two rounds of expert review (*n* = 23). Phase II evaluated the NPET’s psychometric properties by assessing 64 AI-generated nursing care plans based on eight clinical scenarios using eight AI models. A total of 368 individual expert ratings were yielded. Reliability (Cronbach’s α, ICC), content and construct validity (I-CVI, S-CVI/Ave, exploratory factor analysis), and comparative model performance (repeated-measures ANOVA with Tukey post hoc tests) were analyzed.

**Results:**

The NPET demonstrated strong content validity (S-CVI/Ave = 0.88) and excellent internal consistency (α = 0.85–0.94 across domains). Inter-rater reliability was high (ICC_average = 0.85–0.94). Exploratory factor analysis supported the proposed structure: four domains were unidimensional, while the Assessment domain revealed two interpretable factors. Although the overall ANOVA did not reveal statistically significant differences among AI models (F (7, 360) = 1.57, *p* = 0.144, ω² = 0.01), descriptive trends and post hoc tests showed that paid models consistently outperformed free versions. PopAI Paid achieved the highest mean NPET score (M = 3.44 on a 4-point scale), followed by ChatGPT Paid (M = 3.37), while Microsoft Copilot scored the lowest (M = 2.99). The largest pairwise difference—between PopAI Paid and Copilot—yielded a moderate-to-large effect size (Cohen’s d = 0.60).

**Conclusion:**

The NPET is a valid and reliable tool for evaluating the quality of AI-generated nursing care plans. While the overall ANOVA did not yield statistically significant differences across AI models, the consistently high performance across tools and meaningful differences observed in descriptive and post hoc comparisons support the tool’s utility in nursing education, clinical auditing, and AI benchmarking. Future research should explore its application in real-world documentation and monitor its adaptability to evolving AI technologies.

**Supplementary Information:**

The online version contains supplementary material available at 10.1186/s12912-025-04068-8.

## Introduction

The integration of Artificial Intelligence (AI), particularly generative large language models (LLMs), into healthcare represents a paradigm shift with profound implications for nursing practice and education [1, 2]. These technologies promise enhanced efficiency in clinical documentation, improved diagnostic accuracy, and optimized resource allocation [3]. AI-driven systems, such as ChatGPT and Google’s Gemini, are increasingly employed to automate routine tasks, synthesize patient data, and generate preliminary nursing care plans, potentially freeing nurses for higher-value direct patient care [4]. Early evidence supports these benefits: AI applications can reduce nursing documentation time by up to 30%, alleviating administrative burden and enabling nurses to focus more on direct patient care [5]. AI support also appears to improve clinical decision-making; in one survey, over 82% of nurses reported enhanced diagnostic accuracy and expedited clinical judgments with AI assistance, and AI usage was significantly correlated with higher decision accuracy (*p* < 0.05) [5]. These findings align with broader reviews indicating that AI interventions can enhance patient safety by achieving high diagnostic accuracy around 95% with low medication error rates around 1.8% and timely interventions [6].

Despite these potential benefits, significant concerns persist. The variable quality and reliability of AI-generated clinical documentation pose substantial risks to patient safety [1, 7–9]. Studies reveal troubling inconsistencies: Dağcı et al. (2024) found ChatGPT-generated care plans scored only moderately on standard health information metrics, while Haltaufderheide and Ranisch (2024) warn that LLMs often produce superficially coherent but dangerously inaccurate content [10, 11]. Furthermore, comparative studies highlight considerable variability across AI models. Some models outperform others on certain tasks – for instance, excelling in structured queries or multiple-choice diagnostic questions – yet the same models can underperform in more complex clinical reasoning scenarios [12–14]. These inconsistencies suggest that no single AI tool consistently excels across all aspects of nursing documentation, underscoring the need for careful scrutiny of each model’s outputs.

Ensuring that AI-generated nursing documentation meets professional standards requires evaluation criteria aligned with the nursing process (ADPIE) – the five-step framework of Assessment, Diagnosis, Planning, Implementation, and Evaluation that underpins comprehensive, patient-centered nursing care [15, 16]. The ADPIE framework, coupled with NANDA International’s standardized taxonomy of 277 nursing diagnoses, provides the gold standard for structured clinical reasoning and care planning [15]. Adherence to this systematic process is crucial for delivering high-quality, individualized care and for meeting broader patient safety and global health targets [17]. Evaluation of AI outputs in nursing, therefore, should be grounded in these established clinical reasoning standards. However, no validated instrument currently exists to assess the quality of AI-generated nursing care plans across all phases of the ADPIE framework [18]. General-purpose AI evaluation metrics may fail to capture the structured reasoning, clinical nuance, and adherence to taxonomy required in nursing documentation [18]. This gap presents a significant barrier to the safe adoption of AI in nursing practice. Without a rigorous, domain-specific evaluation tool, educators cannot reliably assess student work that has been assisted by AI, clinicians cannot trust AI-generated care plans, and researchers cannot benchmark model performance. Experts emphasize that rigorous validation is “crucial” before deploying generative AI “from text to treatment” [1]. The absence of such an evaluative framework hinders evidence-based integration of AI into nursing workflows, potentially compromising care quality and patient outcomes.

This study aimed to develop and psychometrically validate the Nursing Process Evaluation Tool (NPET), a multidimensional instrument designed to evaluate the integrity of AI-generated nursing documentation. The following research questions guided this work:

1. Does the NPET demonstrate adequate content validity as judged by nursing experts?
2. Does the NPET exhibit strong reliability (internal consistency and inter-rater agreement)?
3. Does the NPET demonstrate construct validity, with items loading onto hypothesized factors?
4. Can the NPET detect significant differences in documentation quality among leading generative AI models?

## Methods

### Study design

This cross-sectional, two-phase study was conducted to develop and validate the Nursing Process Evaluation Tool (NPET) and to compare the documentation quality of leading generative AI models. Phase I involved item generation and content validation using expert consensus.

Phase II assessed the psychometric properties of the NPET and applied it to evaluate AI-generated nursing care plans across standardized scenarios. Ethical approval was granted by the University of Jordan Institutional Review Board, and all participants provided informed consent.

### Instrument development (NPET)

The Nursing Process Evaluation Tool (NPET) was conceptualized, designed, and developed by the research team for the purposes of this study to evaluate the integrity and quality of nursing documentation generated by AI models across all phases of the nursing process. The NPET was designed based on the ADPIE framework (Assessment, Diagnosis, Planning, Implementation, Evaluation) as well as current evidence-based guidelines. It comprises 34 items distributed across five domains: Assessment (11 items), Diagnosis [6], Planning [7], Implementation [5], and Evaluation [5]. Each item is rated on a 4-point rating scale (1 = Not Relevant to 4 = Highly Relevant) and reflects four quality dimensions: accuracy, clinical relevance, completeness, and clarity. Items were derived from established documentation standards, including NANDA-I, and were refined through iterative expert feedback. The full NPET instrument is available in Supplementary File 1.

### Expert panel recruitment

A purposive sample of 23 nursing experts was recruited through professional networks and academic collaborations from four different universities in Jordan (University of Jordan, Yarmouk University, Philadelphia University, and Al-Balqa Applied University), ensuring representation from multiple institutions and specialties. None of the experts participated in the instrument’s development to minimize bias, representing Medical-Surgical (*n* = 5), Maternity/Obstetrics (*n* = 5), Pediatrics (*n* = 5), and Mental Health/Psychiatric Nursing (*n* = 8). Eligibility criteria included a Master’s degree or higher, at least five years of specialty practice, and a current clinical or academic role. Experts participated in both Phase I content validation and Phase II evaluations of AI-generated outputs.

### Content validation (Phase I)

Content validity was established following Polit and Beck’s (2006) guidelines [19]. During Round 1, experts rated each draft item for relevance and clarity using a 4-point scale (1 = not relevant, 2 = somewhat relevant, 3 = quite relevant, and 4 = highly relevant) and provided qualitative feedback [19]. Item-level content validity indices (I-CVI) were calculated as the proportion of experts rating each item as 3 or 4. Items with I-CVI ≥ 0.78 were retained, while those < 0.78 were revised or removed [19]. The scale-level content validity index (S-CVI/Ave) was computed, with ≥ 0.90 considered excellent and ≥ 0.80 acceptable for preliminary validation.

Items were refined for clarity and redundancy, with some merged (e.g., culturally sensitive data gathering was integrated into a holistic assessment item). In Round 2, all 34 retained items achieved I-CVI ≥ 0.78, most exceeding 0.85, resulting in an S-CVI/Ave of 0.88. Face validity was assessed through a 10-point clarity rating across domains, which ranged from 8.0 to 9.7, confirming that the items were clear, relevant, and comprehensive.

### Clinical scenario development (AI outputs)

Eight standardized clinical scenarios were developed to test AI performance: two each from Medical-Surgical, Paediatrics, Maternity, and Mental Health. Each scenario (375–475 words) described patient demographics, medical history, current illness, medications, psychosocial context, physical findings, and relevant diagnostics, aiming to test the models’ ability to identify and address nuanced clinical needs (e.g., cultural considerations, adherence issues, social determinants). Scenarios were constructed by nursing faculty and doctoral students who were not involved in the rating process to minimize bias.

Eight AI models were evaluated: GPT-3.5 and GPT-4o, Gemini Free and Paid, PopAI Free and Paid, Microsoft Copilot, and DeepSeek. All models were prompted identically: *“Develop a comprehensive nursing care plan incorporating assessment*,* NANDA-approved diagnoses*,* planning*,* implementation*,* and evaluation. Ensure interventions are evidence-based and outcomes are measurable.”* This single-turn prompt ensured uniformity across models and minimized prompt engineering variability. In total, 64 AI-generated care plans (8 models × 8 scenarios) were produced, anonymized, and assigned random codes. Experts were blinded to model identity during evaluation. All outputs were saved in standardized text format for structured scoring using the NPET.

### Data collection and rating procedure (Phase II)

Experts rated the AI-generated care plans using the NPET via a secure online platform. Each expert evaluated outputs within their specialty: The Medical–Surgical, Maternity, and Pediatrics outputs were each rated by five experts, whereas the Mental Health outputs were rated by eight experts to capture the domain’s broader subspecialty scope, yielding a total of 368 individual evaluations.

Experts rated each of the 34 NPET items per plan using 4-point rating scale (1 = Very poor/absent, 2 = Limited, 3 = Good, 4 = Excellent), considering accuracy, clinical relevance, completeness, and clarity. rather than on a global judgment of the plan. To ensure scoring reliability, raters completed standardized training using a sample care plan according to NANDA International (NANDA-I) classifications and scoring guide. Item order was randomized to reduce response bias. Rating sessions were conducted asynchronously over two weeks. Experts were instructed not to discuss content during the evaluation period to preserve rating independence.

**Fig. 1:**
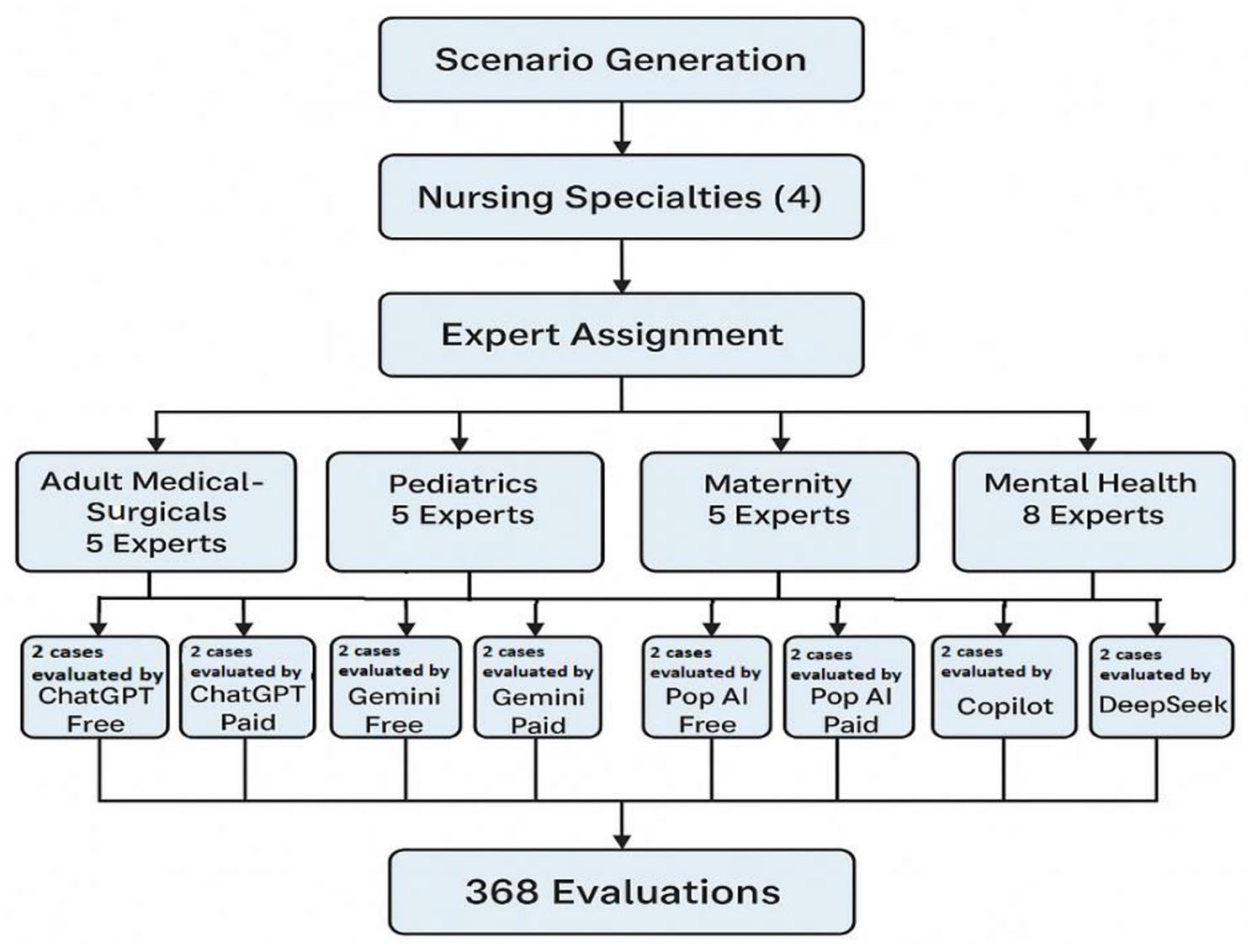
Flowchart of the evaluation design illustrating the process of clinical scenario creation, expert rater allocation, AI tool variations, and total evaluations conducted (*N* = 368)

All AI outputs were anonymized, and raters were blinded to model identity. Figure 1 outlines the overall evaluation process, including scenario creation, AI output generation, and expert assignment.

### Sample size determination

Sample size calculations followed established psychometric recommendations. For exploratory factor analysis (EFA), we used the recommended sample-to-variable ratio (SVR) of 10:1, yielding a minimum of 340 evaluations for 34 items [20]. Accounting for a 10% non-response rate, with 368 valid responses retained after exclusions. For content validation, Polit and Beck (2006) recommend 5–10 experts, but our panel of 23 experts exceeded this standard, improving reliability and generalizability [19].

### Statistical analysis

Analyses were performed using IBM SPSS Statistics v22.0 with a two-tailed significance threshold of α = 0.05. Post hoc power analysis indicated > 90% power to detect medium effect sizes (f ≈ 0.20) under the repeated-measures design with 368 ratings.

#### Content validity

I-CVI and S-CVI/Ave were computed for all items and domains. To complement these, we also calculated scale-level universal agreement (S-CVI/UA) – the proportion of items that all experts rated as relevant (3 or 4). However, S-CVI/UA is a very stringent measure; with 23 experts, requiring universal agreement is likely unrealistic, so we focused on S-CVI/Ave as the primary index [19]. We report the I-CVI for each item, the S-CVI/Ave for each domain (Assessment, Diagnosis, Planning, Implementation, Evaluation), and the instrument-level S-CVI/Ave computed across all items.

#### Reliability

Internal consistency was assessed using Cronbach’s α for each domain of NPET and for the overall scale, with α ≥ 0.70 deemed acceptable and ≥ 0.80 preferred [21]. Inter-rater reliability was estimated via Intraclass Correlation Coefficients (ICC, two-way mixed-effects, consistency type). Average ICCs were calculated per domain and overall, with interpretation thresholds of < 0.50 (poor), 0.50–0.75 (moderate), 0.75–0.90 (good), and > 0.90 (excellent) [22]. Single-rater ICCs were also reported to highlight variability between raters.

#### Construct validity (Exploratory Factor Analysis)

EFA was conducted to examine the factor structure of the NPET to see if items naturally group according to the nursing process domains. Since each domain’s items were designed to capture a single construct, we performed separate EFAs on the items of each domain, rather than one giant EFA on all 34 items (this was a more interpretable approach given the instrument’s intentional subscale structure). Principal Axis Factoring with Promax rotation was applied for each domain’s item set, to allow factors to correlate. Sampling adequacy was assessed via the Kaiser-Meyer-Olkin (KMO) measure (> 0.80 acceptable), and Bartlett’s test of sphericity (*p* < 0.001). Factors with eigenvalues > 1 and loadings > 0.40 were retained. Cross-loading items (> 0.30 on multiple factors) were for potential modification or removal. Given that the NPET was expected to be multidimensional (with at least five domains), we anticipated finding a single dominant factor within each domain’s items. For example, for the Nursing Diagnosis domain’s 6 items, we expected a single factor reflecting “diagnosis quality” – and indeed, a one-factor solution explaining that more than 50% of the variance would support this. If multiple factors emerged in a domain (as happened with Assessment), we interpreted them substantively. We also performed an EFA on the entire scale (all items) to examine the broader factor structure, recognizing that factors might align with specific domains or cut across them (e.g., perhaps an “Accuracy” factor spanning multiple domains). The factor scores from the EFA were saved for use in analyzing model performance, if needed (e.g., to determine whether certain models scored higher on specific factor dimensions). We report variance explained by each factor and any notable cross-loadings or deviations from the hypothesized structure.

#### Comparative analysis of AI tools

Each scenario was treated as a case and compared the NPET scores of different AI models across scenarios. The design is essentially a repeated-measures ANOVA: each scenario was evaluated with each of 8 models, and we have an NPET score (mean item score or domain scores) for each model per scenario. We computed an overall total NPET score for each AI output by summing all item ratings (which range from 34 to 136, as there are 34 items with a maximum of 4 each). Also, domain-specific scores were examined. For the ANOVA, the independent variable was AI Model (within-subjects, eight levels), and the “subjects” were the scenario cases (*N* = 8 scenarios). We first checked the ANOVA assumptions: Mauchly’s test for sphericity and applied Greenhouse-Geisser correction if sphericity was violated (which is common with eight conditions). A one-way repeated measures ANOVA was conducted to determine if there was a significant difference in NPET scores across models. The F statistic, p-value, and effect size (we used ω² for repeated measures, which adjusts for within-subject variance; a value around 0.07, can be interpreted as a moderate effect of the model on scores) were reported. Following a significant ANOVA result, we performed post-hoc pairwise comparisons between models. We used Tukey’s Honest Significant Difference (HSD) test for pairwise contrasts because it controls family-wise error rate well with many comparisons, and we cross-checked with Bonferroni adjustments (they yielded similar significance patterns, with Tukey being slightly more conservative in some cases). For each pair of models, we obtained the mean difference in NPET score, 95% confidence interval, and p-value. We also computed Cohen’s d effect sizes for key pairwise differences, using the pooled standard deviation from the ANOVA residuals to standardize differences. We interpreted d = 0.2 as small, 0.5 medium, 0.8 large effect [23, 24].

All statistical tests were two-tailed. We considered *p* < 0.05 as statistically significant for primary analyses. In post-hoc tests, we applied the Tukey HSD, which inherently adjusts α for multiple comparisons. Thus, any Tukey-adjusted p-value < 0.05 was considered significant. Where relevant, we also mention if a result was marginal (e.g., *p* ~ 0.08) to ensure transparency [23, 24].

### Ethical consideration

All ethical principles were strictly observed to ensure the integrity of the study and the protection of participants. Informed consent was obtained from all expert participants after they were provided with clear information about the study’s purpose, procedures, and their right to withdraw at any stage without penalty. Confidentiality and anonymity were guaranteed throughout the research process; no identifiable information was collected, and all data were securely stored in password-protected files accessible only to the research team.

Ethical approval for the study was granted by the Institutional Review Board (IRB) of Philadelphia University, School of Nursing, Amman, Jordan. Moreover, transparency and honesty in data handling and result reporting were upheld, ensuring that findings were presented objectively, regardless of whether they supported the proposed tool’s effectiveness. Finally, the study was conducted in accordance with the ethical principles of the Declaration of Helsinki and aimed to contribute to the advancement of nursing practice and patient care.

## Results

### Content validity

Table 1 presents the content validity indices (CVI) for the Nursing Process Evaluation Tool (NPET) across its five domains. The overall scale-level CVI (S-CVI/Ave) was 0.88, indicating that, on average, 88% of expert ratings fell into the highest two relevance categories (scores of 3 or 4). All domains surpassed the recommended threshold of 0.80 for acceptable content validity [19].

The Assessment domain (11 items) achieved an S-CVI/Ave of 0.85, with the item “Differentiates subjective vs. objective data” receiving an I-CVI of 0.95. The Nursing Diagnosis domain (6 items) scored 0.84, supported by strong agreement on items such as “Uses correct NANDA-I diagnoses from assessment” (I-CVI = 0.90). Similarly, the Planning domain (7 items) demonstrated an S-CVI/Ave of 0.84, with high ratings for the item “Sets measurable, time-bound goals (SMART)” (I-CVI = 0.87).

The Implementation domain (5 items) exhibited the highest domain-level S-CVI/Ave (0.89), with “Interventions are evidence-based and safe” receiving the strongest consensus (I-CVI = 0.97). The Evaluation domain (5 items) achieved a mean S-CVI/Ave of 0.85, with “Evaluates patient response to interventions” scoring 0.89 on item-level relevance.

Collectively, these results confirm that the NPET domains are highly relevant and aligned with nursing documentation standards, providing a robust foundation for subsequent reliability and validity analyses.

**Table 1:** Content validity indices by domain of the nursing process evaluation tool (NPET)

Nursing Process Domain	Number of Items	Scale-CVI/ Ave	Example Item (with I-CVI)
Assessment	11	0.85	Differentiates subjective vs. objective data – I- CVI 0.95
Nursing Diagnosis	6	0.84	Uses correct NANDA-I diagnoses from assessment – I-CVI 0.90
Planning	7	0.84	Sets measurable, time-bound goals (SMART) – I- CVI 0.87
Implementation	5	0.89	Interventions are evidence-based and safe – I- CVI 0.97
**Evaluation**	5	0.85	Evaluates patient response to interventions – I- CVI 0.89
Overall NPET	34	0.88	– (Overall proportion of relevant ratings)

NPET: Nursing Process Evaluation Tool

S-CVI/Ave: Scale-level Content Validity Index, average method

I-CVI: Item-level Content Validity Index

According to Polit and Beck (2006), I-CVI values ≥ 0.78 and S-CVI/Ave values ≥ 0.80 indicate acceptable content validity for health measurement tools [19]

### Reliability and internal consistency

Table 2 summarizes the internal consistency, inter-rater reliability, and descriptive statistics for the NPET. The overall scale demonstrated excellent internal consistency, with a Cronbach’s α of 0.94, surpassing the ≥ 0.80 benchmark for high-stakes assessment tools [21]. Domain-specific α values ranged from 0.85 (Implementation) to 0.94 (Evaluation), confirming that each domain forms a coherent subscale.

Inter-rater reliability was equally robust. The intraclass correlation coefficient (ICC) for the total NPET score was 0.85 (95% CI: 0.80–0.89), reflecting excellent agreement across expert raters [22]. Domain-level ICCs closely matched Cronbach’s α, with Evaluation (ICC = 0.94, 95% CI: 0.93–0.95) and Planning (ICC = 0.92, 95% CI: 0.91–0.93) showing particularly high inter-rater consistency. Single-rater ICCs were lower, ranging from 0.49 (Diagnosis) to 0.75 (Evaluation), indicating that reliability improves when multiple raters are used.

Descriptive statistics highlight the quality of AI-generated outputs as evaluated by experts. Across all 368 ratings, the mean item score (M) for the NPET was 3.23 (SD = 0.25) on a 1–4 scale, suggesting generally good documentation quality. Among domains, Implementation received the highest average score (M = 3.29, SD = 0.24), while Planning scored slightly lower (M = 3.18, SD = 0.36), indicating relative challenges in defining measurable and time-bound care goals.

These findings confirm that the NPET exhibits strong psychometric properties, with high internal consistency and inter-rater agreement across all domains, ensuring its suitability for evaluating AI-generated nursing documentation.

**Table 2:** Reliability statistics and descriptive summary for NPET (*N* = 368 ratings)

Scale (Domain)	No. of Items	Cronbach’s α	ICC (Avg) [95% CI]	ICC (Single) [95% CI]	Mean Item Score (M)	SD of Summed Domain
Assessment	11	0.92	0.92 [0.90–0.93]	0.50 [0.46–0.54]	3.26	0.33 (Total score range: 11–44)
Nursing Diagnosis	6	0.85	0.85 [0.83– 0.88]	0.49 [0.45–0.54]	3.21	0.27 (Range: 6–24)
Planning	7	0.92	0.92 [0.91– 0.93]	0.63 [0.59–0.67]	3.18	0.36 (Range: 7–28)
Implementation	5	0.85	0.85 [0.82–0.87]	0.53 [0.48–0.58]	3.29	0.24 (Range: 5–20)
Evaluation	5	0.94	0.94 [0.93–0.95]	0.75 [0.71–0.78]	3.20	0.29 (Range: 5–20)
Overall NPET	34	0.94	0.85 [0.80–0.89]	-	3.23	0.25 (average item SD)

NPET: Nursing Process Evaluation Tool

ICC: Intraclass Correlation Coefficient

CI: Confidence Interval

Cronbach’s α ≥ 0.80 indicates strong internal consistency [21]

ICC ≥ 0.75 is considered good, and ≥ 0.90 excellent [22]

Mean Item Score is on a 1–4 scale

SD reflects the standard deviation of summed domain scores unless otherwise noted

### Construct validity: Exploratory Factor Analysis

Exploratory Factor Analysis (EFA) was conducted to evaluate the underlying structure of the NPET and confirm its alignment with the nursing process framework. Sampling adequacy was confirmed for all domains, with Kaiser-Meyer-Olkin (KMO) values exceeding 0.80 and Bartlett’s tests of sphericity significant for each domain (*p* < 0.001), indicating that the data were appropriate for factor analysis.

The Assessment domain (11 items) produced a two-factor solution explaining 58.16% of the total variance. Factor 1, *General Assessment Quality*, accounted for 51.50% of the variance and included items reflecting completeness and clinical relevance. Factor 2, *Data Differentiation* (6.66%), captured the ability to distinguish subjective from objective data, highlighting a distinct subdimension of assessment quality.

The Nursing Diagnosis domain (6 items) showed a unidimensional structure, explaining 50.65% of the variance, with strong loadings on items addressing clinical accuracy and standardized NANDA-I terminology. Similarly, the Planning domain (7 items) demonstrated a single-factor structure, accounting for 63.35% of the variance, with high loadings for items related to evidence-based, measurable care planning.

The Implementation domain (5 items) revealed one dominant factor explaining 53.97% of variance, emphasizing intervention clarity and alignment with best practices. The Evaluation domain (5 items) exhibited the strongest psychometric performance, with a single factor explaining 74.82% of the variance, reflecting consistent evaluation of patient outcomes and response to interventions.

These findings confirm that the NPET domains are conceptually coherent and psychometrically distinct, supporting its multidimensional design. The two-factor structure observed in the Assessment domain further underscores the importance of differentiating general documentation quality from data categorization.

Table 3 summarizes the KMO values, Bartlett’s χ² tests, explained variance, and factor structures for each domain.

**Table 3:** Summary of factor analysis results by nursing process domain

Domain	No. of Items	KMO	Bartlett’s χ² (df) *	Variance Explained	Key Factor Structure
Assessment	11	0.90	2378.77 (55)	58.16%	Two factors: F1: General Assessment Quality (51.50%) F2: Data Differentiation (6.66%)
Nursing Diagnosis	6	0.82	1024.42 (15)	50.65%	Single Factor: Diagnosis Quality
Planning	7	0.92	1698.77 (21)	63.35%	Single Factor: Planning Effectiveness
Implementation	5	0.82	794.83 (10)	53.97%	Single Factor: Implementation Fidelity
Evaluation	5	0.89	1542.23 (10)	74.82%	Single Factor: Evaluation Rigor

KMO: Kaiser–Meyer–Olkin measure of sampling adequacy

χ² (df): Chi-square value with degrees of freedom for Bartlett’s test of sphericity

Variance Explained: Total variance accounted for by retained factor(s)

*All Bartlett’s tests were statistically significant at *p* < 0.001, indicating suitability for factor analysis

### Comparative performance of AI models

**Fig. 2:**
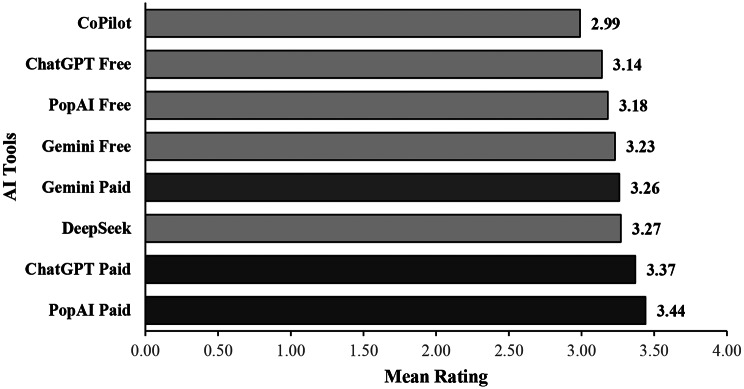
Mean expert ratings for AI tool variants out of 4

Figure 2 displays the mean NPET ratings (1–4 scale) for the eight AI models evaluated. A one-way repeated-measures ANOVA revealed no statistically significant effect of AI model on documentation quality, F(7, 360) = 1.57, *p* = 0.144, with a small effect size (ω² ≈ 0.01).

Despite the non-significant omnibus test, descriptive trends revealed that paid AI tools consistently outperformed their free counterparts. Paid AI tools consistently outperformed their free counterparts. PopAI Paid achieved the highest mean NPET score (M = 3.44, on a 4-point scale), followed closely by ChatGPT Paid (M = 3.37), DeepSeek (M = 3.27), and Gemini Paid (M = 3.26). Free models demonstrated lower performance, with Gemini Free (M = 3.23), PopAI Free (M = 3.18), and ChatGPT Free (M = 3.14) scoring notably below the top-performing tools. Microsoft CoPilot recorded the lowest mean score (M = 2.99). Although the overall model effect was not statistically significant, Tukey HSD post hoc tests revealed several significant pairwise differences. Notably, the performance gap between PopAI Paid and Microsoft Copilot yielded a Cohen’s d = 0.60, indicating a moderate-to-large effect size favoring PopAI Paid. These results suggest that, while the NPET did not statistically differentiate model performance at the omnibus level, there are meaningful practical differences between individual tools—particularly between paid and free AI solutions—when applied to clinical nursing documentation.

### Post hoc comparisons between AI models

To further examine differences in model performance, Tukey’s Honest Significant Difference (HSD) test was conducted following the significant repeated-measures ANOVA. As detailed in Table 4, several statistically significant pairwise differences emerged among the eight AI models, highlighting clear performance stratification.

ChatGPT Paid outperformed CoPilot with a moderate effect size (M difference = 0.383, SE = 0.11, 95% CI [0.13, 0.64], *p* = 0.002, d = 0.51). Likewise, PopAI Paid was rated significantly higher than PopAI Free (M difference = 0.26, SE = 0.11, 95% CI [0.01, 0.51], *p* = 0.039, d = 0.35), and ChatGPT Free was significantly lower than PopAI Paid (M difference = – 0.30, SE = 0.11, 95% CI [–0.55, − 0.05], *p* = 0.018, d = – 0.40).

Interestingly, CoPilot was also rated significantly lower than PopAI Paid (M difference = – 0.45, SE = 0.12, 95% CI [–0.72, − 0.17], *p* < 0.001, d = – 0.60), reinforcing the model’s comparatively weaker performance.

Other pairwise comparisons did not reach statistical significance (*p* > 0.05), though some revealed moderate effect sizes. For instance, the difference between CoPilot and Gemini Paid approached significance (*p* = 0.082, d = – 0.35), suggesting potential trends worthy of further exploration.

**Table 4:** *Tukey HSD pairwise comparisons of AI tool ratings (*N* = 46 per tool)

Comparison	Mean Difference	SE	95% CI	*p*-value	Cohen’s d
ChatGPT Free vs. ChatGPT Paid	-0.24	0.11	[-0.49, 0.02]	0.092	-0.32
ChatGPT Free vs. CoPilot	0.15	0.12	[-0.14, 0.43]	0.558	0.19
ChatGPT Free vs. DeepSeek	-0.12	0.11	[-0.37, 0.13]	0.754	-0.16
ChatGPT Free vs. Gemini Free	-0.11	0.11	[-0.35, 0.14]	0.825	-0.14
ChatGPT Free vs. Gemini Paid	-0.12	0.11	[-0.36, 0.12]	0.766	-0.16
ChatGPT Free vs. PopAI Free	-0.04	0.11	[-0.29, 0.21]	0.992	-0.06
**ChatGPT Free vs. PopAI Paid**	-0.30	0.11	**[-0.55**,** -0.05]**	**0.018**	-0.40
**ChatGPT Paid vs. CoPilot**	0.38	0.11	**[0.13**,** 0.64]**	**0.002**	0.51
ChatGPT Paid vs. DeepSeek	0.12	0.11	[-0.13, 0.36]	0.837	0.16
ChatGPT Paid vs. Gemini Free	0.13	0.11	[-0.11, 0.38]	0.702	0.18
ChatGPT Paid vs. Gemini Paid	0.12	0.11	[-0.12, 0.36]	0.758	0.16
ChatGPT Paid vs. PopAI Free	0.20	0.11	[-0.05, 0.45]	0.280	0.26
ChatGPT Paid vs. PopAI Paid	-0.06	0.110	[-0.31, 0.18]	0.956	-0.08
CoPilot vs. DeepSeek	-0.27	0.12	[-0.55, 0.01]	0.064	-0.36
CoPilot vs. Gemini Free	-0.25	0.12	[-0.53, 0.03]	0.096	-0.33
CoPilot vs. Gemini Paid	-0.26	0.12	[-0.54, 0.01]	0.082	-0.35
CoPilot vs. PopAI Free	-0.19	0.12	[-0.46, 0.09]	0.380	-0.25
**CoPilot vs. PopAI Paid**	-0.45	0.12	**[-0.72**,** -0.17]**	**< 0.001**	-0.60
DeepSeek vs. Gemini Free	0.02	0.11	[-0.23, 0.26]	0.999	0.02
DeepSeek vs. Gemini Paid	0.00	0.11	[-0.24, 0.25]	1.000	0.00
DeepSeek vs. PopAI Free	0.08	0.11	[-0.17, 0.33]	0.941	0.11
DeepSeek vs. PopAI Paid	-0.18	0.11	[-0.42, 0.06]	0.318	-0.24
Gemini Free vs. Gemini Paid	-0.02	0.11	[-0.26, 0.23]	0.999	-0.02
Gemini Free vs. PopAI Free	0.06	0.11	[-0.18, 0.31]	0.970	0.09
Gemini Free vs. PopAI Paid	-0.20	0.11	[-0.44, 0.05]	0.280	-0.26
Gemini Paid vs. PopAI Free	0.08	0.11	[-0.17, 0.33]	0.944	0.11
Gemini Paid vs. PopAI Paid	-0.18	0.11	[-0.43, 0.06]	0.340	-0.24
**PopAI Free vs. PopAI Paid**	-0.26	0.11	**[-0.51**,** -0.01]**	**0.039**	-

1. **Pooled SD** = 0.748 (used for Cohen’s d

2. **Cohen’s d** = Mean Difference / Pooled SD

3. **Bold** indicates statistical significance (*p* < 0.05)

4. SE = Standard Error = √(MSwithin/n) = √(0.507/42) ≈ 0.110

5. 95% CI = Mean Difference ± (q-critical × SE)

6. Effect Size Interpretation:

• d = 0.20: Small

• d = 0.50: Medium

• d = 0.80: Large

## Discussion

The Nursing Process Evaluation Tool (NPET) was developed to assess the quality of AI-generated nursing care plans across the ADPIE framework. In this study, NPET demonstrated strong psychometric performance. Expert review showed very high item relevance (item-level CVIs ≥ 0.79 and overall S-CVI/Ave = 0.88), indicating excellent content validity. The tool also exhibited high reliability: internal consistency was robust (Cronbach’s α = 0.85–0.94 across domains, α = 0.94 overall), and inter-rater agreement was strong (ICC_average ≈ 0.85–0.94); supporting the tool’s capacity for consistent evaluation across different raters. Factor analyses largely confirmed the intended structure: each nursing process domain formed a coherent factor (single dominant factor per domain, except Assessment split into “general quality” and “data differentiation”). Finally, NPET scores distinguished among AI models in expected ways, with fine-tuned, larger models (e.g. paid versions of ChatGPT and PopAI) significantly outperforming smaller or free models. In summary, NPET appears to be a valid, reliable instrument for auditing AI-generated nursing documentation. Below, we discuss the findings according to the research questions (RQ1–RQ4) and their implications.

### Content validity (RQ1)

Experts reached near-unanimous consensus on the relevance of NPET items, with I-CVI values ≥ 0.79 (most > 0.85) and a scale-level CVI (S-CVI/Ave) of 0.88 after two review rounds. In practical terms, the S-CVI/Ave of 0.88 indicates that, on average, 88% of experts rated each item as ‘quite’ or ‘highly’ relevant. Such agreement is comparable to other validated nursing documentation tools; for example, the Italian Q-DIO R achieved similarly strong expert endorsement across its items [25].

The NPET’s content validity approaches the 0.90 benchmark often recommended for new instruments [19], supporting its comprehensiveness and relevance.

The strong expert consensus can be attributed to the tool’s grounding in standardized nursing frameworks such as NANDA-I diagnoses, NIC interventions, and NOC outcomes. Experts particularly endorsed the domains of Accuracy, Clinical Relevance, Completeness, and Clarity/Readability as essential for evaluating nursing care plans. These dimensions align with established guidelines emphasizing accurate, comprehensive, and patient-centered documentation [26]. For instance, standardized languages like NANDA-I and NIC provide structured conceptual frameworks, which are reflected in NPET’s organized item set [26].

Overall, these findings confirm that NPET content adequately captures the core criteria of high-quality nursing documentation, consistent with theoretical foundations and prior instruments in the field.

### Reliability (RQ2): internal consistency and inter-rater reliability of the NPET

The NPET demonstrated excellent reliability, encompassing both internal consistency and inter-rater agreement—key attributes for robust application in clinical and research settings. Cronbach’s α values ranged from 0.85 (Implementation) to 0.94 (Evaluation), with the overall 34-item scale achieving α = 0.94. These coefficients surpass the widely accepted threshold of 0.70 for acceptable reliability and compare favorably to established nursing documentation tools such as the Q-DIO-R [25]. Strong item-total correlations further confirm that each domain measures a coherent and distinct dimension of nursing process quality without being disproportionately influenced by individual items.

Inter-rater reliability was equally strong, with ICC values ranging from 0.85 to 0.94 across domains, and three domains (Assessment, Planning, and Evaluation) exceeding 0.90, indicating excellent agreement [22]. While single-rater ICCs were lower (0.49–0.75)—an expected outcome given subjective variations in individual scoring—Evaluation remained highly reliable (ICC ≈ 0.75). These findings highlight NPET’s capacity to deliver consistent, repeatable assessments of care plan quality across raters.

From a practical standpoint, such reliability strengthens the NPET’s suitability for benchmarking the integrity of AI-generated nursing documentation, where inter-rater consistency is crucial for fair model comparisons. It also positions the tool as a valuable instrument for nursing education and clinical audits, enabling standardized evaluation of care plans while supporting quality improvement initiatives.

### Construct validity (RQ3): factor analysis of instrument structure

The factor structure of the NPET largely confirmed its theoretical alignment with the nursing process framework (ADPIE). Four domains—Diagnosis, Planning, Implementation, and Evaluation—exhibited unidimensionality, with strong factor loadings (λ = 0.71–0.89) and substantial explained variance (50–75%). This supports the instrument’s conceptual integrity, indicating that these domains reliably capture cohesive constructs such as “diagnostic accuracy” or “planning effectiveness” [26].

The Assessment domain revealed a more nuanced structure, splitting into General Assessment Quality (51.5% variance) and Data Differentiation (6.7%), reflecting the distinct skill of separating subjective and objective findings. Clinically, this is consistent with the complexity of real-world assessment processes, and it highlights areas where both AI models and nurses require precision. The absence of problematic cross-loadings reinforces the clarity of the instrument’s structure, though minor refinements—such as revising a low-communality item (≈ 0.39)—could enhance future versions. These results are in line with recent psychometric evaluations of nursing documentation tools [25] and confirm NPET’s suitability for evaluating both human- and AI-generated care plans.

### AI model discrimination (RQ4): comparative performance of AI models

Our findings indicate that the Nursing Process Evaluation Tool (NPET) did not statistically differentiate performance among AI tools, as the overall ANOVA was non-significant; nevertheless, the overall performance of the AI tools in generating nursing care plans was generally high, with most models achieving mean NPET scores almost above 3 on a 4-point scale. However, descriptive trends and pairwise comparisons still revealed meaningful differences that warrant discussion. Paid models consistently outperformed free versions, with PopAI and ChatGPT demonstrating the strongest alignment with nursing process standards. This trend reflects the broader trajectory of advanced AI systems, where larger architectures (e.g., GPT-4) and domain-specific fine-tuning contribute to improved narrative coherence, accuracy, and clinical relevance. Conversely, models like Microsoft Copilot, which are optimized for task completion and web-based queries, underperformed when evaluated on structured, documentation-intensive tasks such as nursing care planning.

These findings align with the emerging literature on AI performance in clinical and educational settings, studies have shown that GPT-4-based tools outperform earlier generations, particularly in reasoning-intensive and open-text tasks [18, 27]. Similarly, Mahmood et al. (2024) reported that ChatGPT achieved higher accuracy in nursing-related Q&A tasks compared to other models such as Bard [13]. Our study extends these observations by demonstrating that these performance gaps translate to the generation of comprehensive care plans that adhere to the ADPIE framework. The superior performance of PopAI Paid likely reflects its targeted fine-tuning for healthcare documentation, enabling better integration of standardized nursing languages such as NANDA-I.

The competitive performance of DeepSeek is particularly noteworthy given its status as a free, lesser-known model. Recent evaluations have highlighted DeepSeek’s strong medical reasoning and diagnostic capabilities, sometimes rivaling GPT-4-based systems [28, 29]. Our findings reinforce its potential as a cost-effective alternative for institutions seeking AI tools for clinical documentation without incurring high licensing costs.

Interestingly, our results diverge from some prior assessments of Microsoft Copilot. Previous studies, such as those by Aksoy and Arslan (2025), found Copilot to excel in multiple-choice exam contexts, sometimes outperforming ChatGPT-4 [14]. This discrepancy highlights the importance of task specificity in AI benchmarking: while Copilot’s concise, retrieval-based responses may excel in fact-checking tasks, it struggles with the integrative narrative structure required for nursing care plans. This reinforces the need for evaluation frameworks like NPET that are tailored to the complexities of clinical documentation rather than general Q&A performance.

The findings also underscore that no single model excels universally. While paid models like PopAI and ChatGPT performed strongly, none achieved a perfect NPET score, and each exhibited unique weakness. These observations are consistent with studies reporting that AI models often complement rather than replace each other’s strengths [12]. For nursing practice, this implies that AI-generated documentation should be viewed as a draft or augmentation tool, with nurses providing critical oversight to ensure accuracy, completeness, and clinical appropriateness.

From a practical perspective, these results have significant implications for healthcare settings and nursing education. High-performing models like PopAI Paid can reduce documentation burden by generating drafts that require minimal editing, thereby freeing nurses to focus on direct patient care. Conversely, lower-performing models, such as Copilot, may introduce inefficiencies if their outputs require extensive revision. This is consistent with findings from Thakur and Kashyap’s (2025) meta-analysis, which emphasizes that AI improves diagnostic and safety outcomes only when outputs are accurate, contextually relevant, and carefully validated [30].

Finally, the results highlight the necessity of ongoing benchmarking and refinement of AI tools in healthcare. As models evolve, evaluation frameworks like NPET will play a crucial role in ensuring that AI integration prioritizes safety, quality, and professional accountability. While AI can enhance efficiency and standardization, it cannot replace the nuanced judgment and ethical responsibility of nurses. Therefore, optimal use of these technologies lies in a hybrid approach, where AI assists but does not replace the clinician’s expertise.

### Limitations

This study has several limitations. First, the expert panel was relatively small (*n* = 23) and assessed a limited number of AI-generated outputs (64 across eight models and eight scenarios), which may limit the generalizability of the findings. Including a broader group of stakeholders, such as clinical nurses or nurse educators, could yield different content validity index (CVI) or intraclass correlation coefficient (ICC) estimates.

Second because expert reviewers were aware that they were evaluating AI-generated documentation, there is a possibility of rater bias.

Third, the use of standardized simulated clinical vignettes—rather than real electronic health record (EHR) data—ensured methodological consistency and privacy but reduced ecological validity. Simulated cases cannot fully capture the complexity, variability, and data quality issues present in real-world documentation. Future studies should validate NPET using actual EHR data to confirm its applicability in clinical settings.

Finally, the study’s scope and timing present further constraints. AI models were evaluated at a single point (mid-2025), and their performance may evolve with subsequent updates. Additionally, the tool was tested only on nursing care plans in simulated contexts, so findings may not generalize to other documentation tasks (e.g., progress notes or shift reports) or nursing domains. Future research should examine NPET’s performance across diverse clinical settings, specialties, and AI platforms to enhance its external validity and relevance.

### Implications and future work

#### Practice

The NPET offers a practical rubric for healthcare educators and administrators to audit the quality of AI-assisted nursing documentation. In environments adopting AI tools, NPET can flag deficits (e.g. missing nursing diagnoses or unsupported interventions) that warrant clinician review. By quantifying documentation quality, NPET can guide training: for example, identify common deficits (such as incomplete social histories or vague goals) so that nurses can focus on these areas. This aligns with the broader goal of ensuring AI enhances care without compromising safety. Ultimately, using NPET in routine chart reviews could improve care plan quality and patient outcomes. As AI “increases efficiency” but still needs refinement in accuracy [31], NPET serves as a check – it highlights where AI outputs fall short of professional standards so that models or users can be corrected before patient care is impacted.

#### Research

Future studies should extend validation of the NPET in varied contexts. Additional specialties (e.g. critical care, community health) and settings (acute vs. ambulatory) may raise different documentation priorities. Testing NPET on actual clinical notes (not just scenario-based plans) will assess its usability in real workflows. Moreover, longitudinal research should examine NPET’s sensitivity to improvements in AI: for example, do GPT-5-based plans achieve higher NPET scores than current models? Can NPET detect meaningful gains as models are fine-tuned on nursing curricula? Another avenue is to refine NPET’s factor structure by confirmatory factor analysis in new samples, possibly collapsing items or adding new ones (e.g. specific to interprofessional communication). Researchers should also explore NPET’s predictive validity: does a high NPET score on an AI plan actually correlate with better learner outcomes or safer care in practice? Finally, as LLMs evolve or as new AI architectures emerge (e.g. domain-specific models), the NPET itself may need updating. As Aksoy and colleagues note, AI accuracy depends on model version and task complexity [14]. Ongoing validation will ensure NPET remains aligned with current technology.

## Conclusion

The newly developed NPET demonstrates strong preliminary psychometric properties for evaluating AI-generated nursing care plans in simulated settings. While further validation in real-world clinical contexts is warranted, the tool received strong expert endorsement, yielded reliable scores, and aligned well with the theoretical structure of the nursing process.

Although the overall ANOVA did not yield statistically significant differences in performance among AI models, descriptive trends and pairwise comparisons revealed meaningful differences—particularly the stronger performance of paid, fine-tuned models compared to free or general-purpose tools.

These findings generally support prior research suggesting that more advanced or domain-specific models generate more coherent and clinically appropriate documentation. However, they also highlight the context-dependent nature of “AI performance” and the importance of aligning tool selection with task requirements.

AI tools hold substantial promise to augment nursing practice—enhancing documentation efficiency, consistency, and potentially patient outcomes. Yet, realizing this promise requires thoughtful integration, continuous evaluation, and ethical oversight. The NPET can serve as a valuable benchmarking instrument in this process, helping healthcare educators and institutions make informed, evidence-based decisions about AI adoption while upholding the quality and integrity of patient care.

## Supplementary Information

Below is the link to the electronic supplementary material.

Supplementary Material 1

## Data Availability

No datasets were generated or analysed during the current study.
