# Supplementary material for: Development and validation of the Nursing Process Evaluation Tool (NPET): a multidimensional instrument for assessing the quality of AI-generated nursing documentation
Source: BMC Nurs. 2025 Nov 21;24:1422. doi: 10.1186/s12912-025-04068-8 (PMC12639769; doi:10.1186/s12912-025-04068-8)
Supplement: Supplementary file 1 — Supplementary Material 1 [file 12912_2025_4068_MOESM1_ESM.docx]

**Nursing Process Evaluation Tool**

**Rating Scale:**

1 = Not Relevant. 2 = Somewhat Relevant. 3 = Quite Relevant. 4 = Highly Relevant

| **Nursing Process Component** | **Accuracy** | **4** | **3** | **2** | **1** |
| --- | --- | --- | --- | --- | --- |
| **Assessment** | 1. Responses must be factually correct according to established nursing guidelines and evidence-based practices. |  |  |  |  |
|  | 1. Data must be validated and systematically organized to ensure correctness. |  |  |  |  |
|  | 1. Differentiation between subjective vs. objective data |  |  |  |  |
|  | 1. Accurately identifies priority health issues and concerns. |  |  |  |  |
|  | **Clinical Relevance** | **4** | **3** | **2** | **1** |
|  | 1. The tool must consider a holistic approach (physical, psychological, social, and spiritual aspects). |  |  |  |  |
|  | 1. Responses should align with the patient’s condition and healthcare setting. |  |  |  |  |
|  | 1. Include a cultural competence check to assess patient diversity considerations |  |  |  |  |
|  | **Completeness** | **4** | **3** | **2** | **1** |
|  | 1. The AI tool must collect and analyze all relevant patient data (subjective and objective). |  |  |  |  |
|  | 1. All important details and considerations should be included e.g., historical health data, allergies, and risk factors. |  |  |  |  |
|  | **Clarity and Readability** | **4** | **3** | **2** | **1** |
|  | 1. Data should be clear, structured, and easy to interpret for both healthcare professionals and patients. |  |  |  |  |
|  | 1. Clearly describes the patient's health status based on gathered data (e.g., physical examination, patient history, diagnostic tests). |  |  |  |  |
|  |  |  |  |  |  |
| **Nursing Diagnosis** | **Accuracy** | **4** | **3** | **2** | **1** |
|  | 1. Diagnoses should be accurate and derived directly from assessment data. |  |  |  |  |
|  | 1. The tool must use standardized nursing terminology (e.g., NANDA-I). |  |  |  |  |
|  | **Clinical Relevance** | **4** | **3** | **2** | **1** |
|  | 1. Diagnoses must be clinically relevant to the patient’s condition and aligned with the nursing process. |  |  |  |  |
|  | 1. Diagnoses should be prioritized correctly for appropriate interventions. |  |  |  |  |
|  | **Completeness** | **4** | **3** | **2** | **1** |
|  | 1. The tool must ensure comprehensive identification of all applicable diagnoses. |  |  |  |  |
|  | **Clarity and Readability** | **4** | **3** | **2** | **1** |
|  | 1. Terminology should be clear, standardized, and easy to understand by nurses and other healthcare professionals. |  |  |  |  |

| **Nursing Process Component** | **Accuracy** | **4** | **3** | **2** | **1** |
| --- | --- | --- | --- | --- | --- |
| **Planning** | 1. Goals must be SMART (Specific, Measurable, Achievable, Relevant, Time-bound). |  |  |  |  |
|  | 1. Interventions must be evidence-based and factually correct. |  |  |  |  |
|  | **Clinical Relevance** | **4** | **3** | **2** | **1** |
|  | 1. The care plan must be tailored to the patient’s needs, condition, and healthcare setting. |  |  |  |  |
|  | 1. The tool must support collaboration with the healthcare team and patient to enhance patient-centered care. |  |  |  |  |
|  | **Completeness** | **4** | **3** | **2** | **1** |
|  | 1. The plan should cover all necessary aspects of patient care, ensuring a comprehensive approach. |  |  |  |  |
|  | 1. Ensure short-term and long-term goals are considered rather than only immediate interventions |  |  |  |  |
|  | **Clarity and Readability** | **4** | **3** | **2** | **1** |
|  | 1. Goals and interventions must be clearly stated and well-structured to facilitate implementation by the care team. |  |  |  |  |
|  |  |  |  |  |  |
| **Implementation** | **Accuracy** | **4** | **3** | **2** | **1** |
|  | 1. All interventions must be accurate, safe, and timely, adhering to clinical guidelines and evidence-based practices. |  |  |  |  |
|  | **Clinical Relevance** | **4** | **3** | **2** | **1** |
|  | 1. The tool must respect patient preferences and cultural values. |  |  |  |  |
|  | 1. Actions must be aligned with best nursing practices for the given healthcare setting (hospital, home health, outpatient, etc.). |  |  |  |  |
|  | **Completeness** | **4** | **3** | **2** | **1** |
|  | 1. The tool should ensure that all necessary interventions and patient responses are documented. |  |  |  |  |
|  | **Clarity and Readability** | **4** | **3** | **2** | **1** |
|  | 1. Actions and documentation should be concise, structured, and easy to interpret for continuity of care. |  |  |  |  |
|  |  |  |  |  |  |
| **Evaluation** | **Accuracy** | **4** | **3** | **2** | **1** |
|  | 1. The AI tool must measure patient progress against expected outcomes and ensure that assessments are factually correct. |  |  |  |  |
|  | 1. Effectiveness of interventions should be analyzed using evidence-based nursing practices. |  |  |  |  |
|  | **Clinical Relevance** | **4** | **3** | **2** | **1** |
|  | 1. Adjustments should be made to the care plan based on evaluation findings to maintain clinical relevance and improve patient outcomes. |  |  |  |  |
|  | **Completeness** | **4** | **3** | **2** | **1** |
|  | 1. The evaluation must comprehensively assess all aspects of patient progress, ensuring nothing is overlooked. |  |  |  |  |
|  | **Clarity and Readability** | **4** | **3** | **2** | **1** |
|  | 1. Evaluation findings must be clearly documented and communicated to ensure transparency and ease of use. |  |  |  |  |
